# *CYP2D6* copy number determination using digital PCR

**DOI:** 10.3389/fphar.2024.1429286

**Published:** 2024-08-14

**Authors:** Wendy Y. Wang, Lancy Lin, Erin C. Boone, Junko Stevens, Andrea Gaedigk

**Affiliations:** ^1^ Division of Clinical Pharmacology, Toxicology and Therapeutic Innovation, Children’s Mercy Research Institute (CMRI), Kansas City, MO, United States; ^2^ Genetic Sciences Division, Thermo Fisher Scientific, Waltham, CA, United States; ^3^ School of Medicine, University of Missouri-Kansas City, Kansas City, MO, United States

**Keywords:** CYP2D6, copy number variation, digital PCR, multiplex, One-pot, Absolute Q, quantitative PCR, reference gene

## Abstract

**Background:**

*CYP2D6* testing is increasingly used to guide drug therapy and thus, reliable methods are needed to test this complex and polymorphic gene locus. A particular challenge arises from the detection and interpretation of structural variants (SVs) including gene deletions, duplications, and hybrids with the *CYP2D7* pseudogene. This study validated the Absolute Q^™^ platform for digital PCR-based *CYP2D6* copy number variation (CNV) determination by comparing results to those obtained with a previously established method using the QX200 platform. In addition, protocols for streamlining *CYP2D6* CNV testing were established and validated including the “One-pot” single-step restriction enzyme digestion and a multiplex assay simultaneously targeting the *CYP2D6* 5′UTR, intron 6, and exon 9 regions.

**Methods:**

Genomic DNA (gDNA) samples from Coriell (n = 13) and from blood, saliva, and liver tissue (n = 17) representing 0–6 copies were tested on the Absolute Q and QX200 platforms. Custom TaqMan™ copy number (CN) assays targeting *CYP2D6* the 5′UTR, intron 6, and exon 9 regions and a reference gene assay (*TERT* or RNaseP) were combined for multiplexing by optical channel. In addition, two digestion methods (One-pot digestion and traditional) were assessed. Inconclusive CN values on the Absolute Q were resolved using an alternate reference gene and/or diluting gDNA.

**Results:**

Overall, results between the two platforms and digestions methods were consistent. The “One-pot” digestion method and optically multiplexing up to three *CYP2D6* regions yielded consistent result across DNA sample types and diverse SVs, reliably detecting up to 6 gene copies. Rare variation in reference genes were found to interfere with results and interpretation, which were resolved by using a different reference.

**Conclusion:**

The Absolute Q produced accurate and reliable *CYP2D6* copy number results allowing for a streamlined and economical protocol using One-pot digestion and multiplexing three target regions. Protocols are currently being expanded to other pharmacogenes presenting with SVs/CNVs.

## 1 Introduction

*CYP2D6* is a highly polymorphic gene encoding the cytochrome P450 2D6 enzyme, which contributes to the metabolism and bioactivation of many prescribed medications (Saravanakumar et al., 2019). The Clinical Pharmacogenomics Consortium (CPIC) has published guidelines for *CYP2D6* gene-drug pairs, underscoring the relevance and importance of this gene in clinical settings (Hicks et al., 2015; Bell et al., 2017; Hicks et al., 2017; Goetz et al., 2018; Brown et al., 2019; Crews et al., 2021; Bousman et al., 2023; Duarte et al., 2024). Since CYP2D6 is involved in the metabolism of over 20% of clinically prescribed drugs (Saravanakumar et al., 2019), understanding *CYP2D6* variation is important for guiding drug therapy. Currently the Pharmacogene Variation Consortium (PharmVar), which collects, curates, and standardizes nomenclature of important pharmacogenes (Gaedigk et al., 2021; Turner et al., 2023), lists over 160 distinct star (*) alleles for *CYP2D6*.

A myriad of single nucleotide variants (SNVs) contributes to the observed range in CYP2D6 activity across individuals and populations, and structural variants (SVs) add another layer of complexity to the highly polymorphic gene locus. SVs include gene duplications and multiplications, gene deletions (CYP2D6*5), and hybrid gene copies with the *CYP2D7* pseudogene (CYP2D6*13, *36, *68, etc.). These are also referred to as copy number variants (CNVs). Since the presence of these SVs/CNVs also affects a patient’s phenotype (metabolizer status) and therapeutic decision making, it is imperative that SVs/CNVs are accurately detected. Accordingly, the Association for Molecular Pathology (AMP) recommends that clinical *CYP2D6* genotyping include CNV testing as part of both Tier 1 and Tier 2-level testing (Pratt et al., 2021). A PharmVar tutorial on *CYP2D6* structural variation (Turner et al., 2023) provides an overview of methods and strategies for SV/CNV testing including sequence-based and targeted detection-based methods such as arrays, quantitative PCR (qPCR), digital PCR (dPCR) and mass spectrometry-based approaches, with discussions of challenges and pitfalls. Additionally, the PharmVar “Structural Variation” document (available at https://www.pharmvar.org/gene/CYP2D6) details currently known SVs/CNVs and provides recommendations for reporting.

**FIGURE 1:**
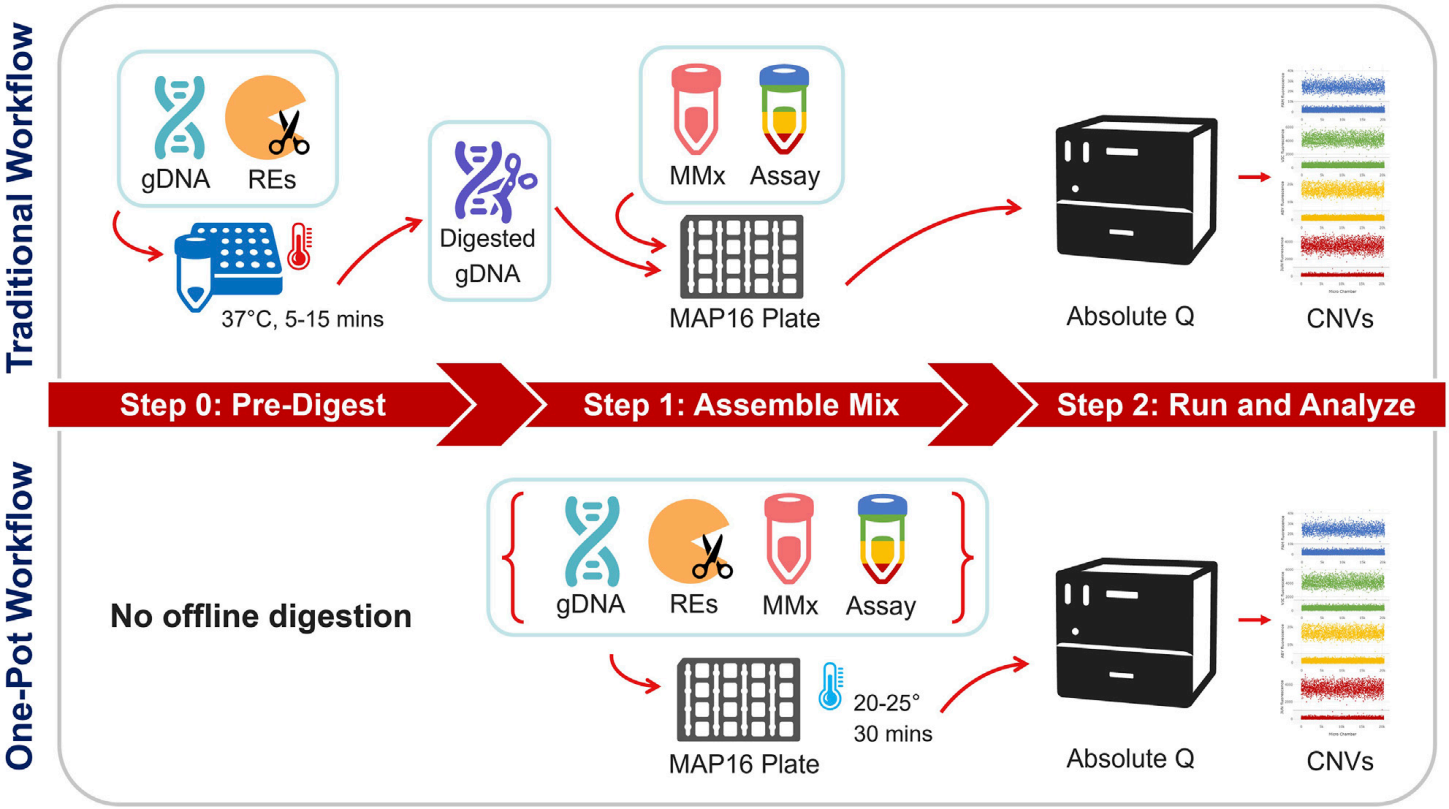
Overview of the traditional vs. One-pot workflows for dPCR copy number testing. In the traditional workflow, gDNA samples undergo a pre-work step via restriction enzyme (RE) digestion at 37°C. Digested gDNA is then incorporated into a reaction mixture containing the dPCR master mix and multiplex assay. The assembled mixture and isolation buffer are loaded onto a MAP16 plate and subsequently run on the Absolute Q dPCR instrument. The One-pot workflow contrasts by skipping the pre-work step and directly incorporating the RE into the reaction mixture. After loading the assembled mixture and isolation buffer, the MAP16 plate incubates at ambient laboratory conditions before being transferred to the dPCR instrument.

Digital PCR is a methodology that enables the absolute quantification of targets which is superior to qPCR’s relative quantification, which is also known as the comparative Ct method (Schmittgen and Livak, 2008). Absolute quantification has been shown to not only be more robust, reproducible, and sensitive but also allows discrimination of higher copy number states and does not require external CN standards for run validation (Hindson et al., 2011). For these reasons, along relatively rapid turn-around times, dPCR is well suited especially for testing in clinical settings (Gaedigk et al., 2019; Zhang et al., 2022; Turner et al., 2023). In contrast to qPCR, which measures the amplification of one target and reference assay in a single reaction, dPCR platforms disperse the reaction mixes into ≥20,000 microreactions for quantification of both the target and reference assays, enabling the sensitive detection of low concentration analytes. This can be achieved in different ways. The Bio-Rad QX200™ Digital PCR System disperses reaction mixes by oil emersion and microfluidics in a propriety method termed “droplet digital PCR” (ddPCR™) (Hindson et al., 2011). The Thermo Fisher Scientific (TFS) Applied Biosystems™ QuantStudio™ Absolute Q™ dPCR System utilizes a microfluidic array plate (MAP16 plate) for compartmentalizing into microchambers (Dueck et al., 2019). An overview of the Absolute Q dPCR workflow is provided in Figure 1. Microreactions are subsequently thermocycled and fluorescent signals are detected (binary readings of either present or absent). The Poisson distribution is then applied to statistically approximate the concentration of each target. In short, dPCR allows for CN determination by comparing the concentration (copies/µL) of the “positive” microreactions of unknown targets to the concentration of the “positive” reactions of the reference gene. Multiplying this ratio by the number of expected reference gene copies, usually a 2-copy reference, provides the “calculated” CN value. This calculation is typically performed by the platform’s software and does not require further analysis steps for CN interpretation (Quan et al., 2018).

Although qPCR is widely being used for pharmacogenetic copy number testing, there are some disadvantages. Results from qPCR can be more sensitive to the quality and purity of the input genomic DNA because they are dependent on a real-time signal from one reaction, as opposed to the endpoint signal of up to 20,000 microreactions with dPCR. Depending on concentration and type of inhibitor(s) present in a DNA sample, the overall qPCR amplification signal may be affected, whereas there is a decreased reliance on amplification kinetics for endpoint dPCR quantification. Furthermore, inhibitors may be compartmentalized into a portion of dPCR microreactions, decreasing the interactions between the inhibitor molecules and PCR reagents (Quan et al., 2018; Sidstedt et al., 2020). Thus, for qPCR, sub-optimal quality of a DNA preparation may lead to greater differences in amplification efficiency, which can result in ambiguous or incorrect CN calls. Another disadvantage of qPCR is the requirement of CN control samples to correlate Ct values into copy number calls for each plate run. In dPCR, CN controls may be included to assess performance, however they are not necessary to determine a copy number call because CN is calculated from the ratio of targeted copies. qPCR also requires technical replicates (triplicates or even quadruplicates) to ensure call confidence. Furthermore, higher copy number states are not easily resolved using qPCR due to assay limitations and are typically reported as ≥3. Moreover, dPCR has the advantage that two or more unknown targets can be multiplexed for time and/or cost savings while each gene target is run separately for qPCR (Whale et al., 2016).

**FIGURE 2:**
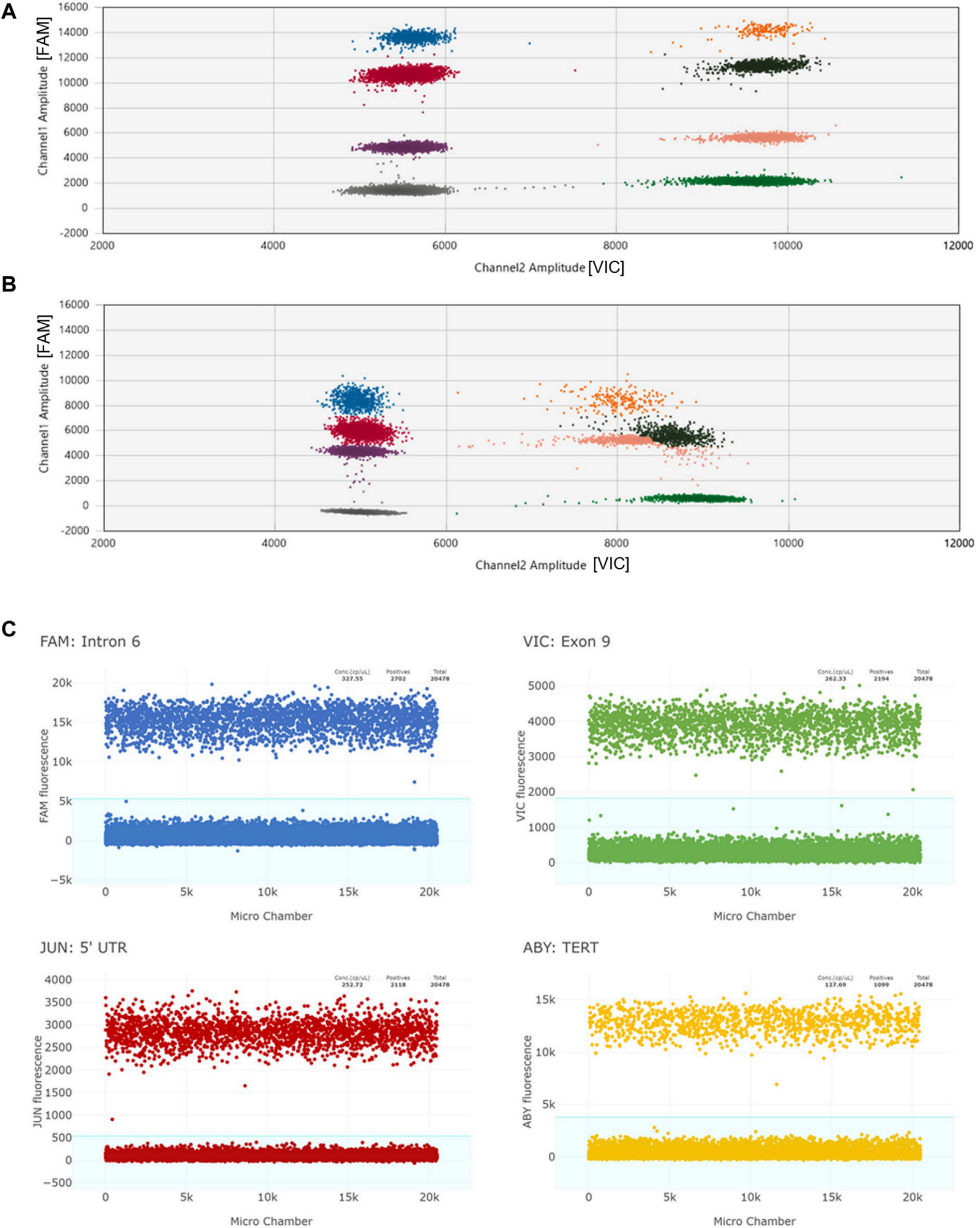
Panel **(A)** depicts an example of a 2D scatter plot when multiplexing by amplitude using the QX200 ddPCR system. Individual clusters are well-defined. “Channel 1” plots FAM reactions and “Channel 2” plots VIC reactions. The two *CYP2D6* targets are labelled with FAM, and the reference gene target is labelled with VIC. Panel **(B)** represents a 2D scatter plot when multiplexing by amplitude under suboptimal conditions (likely due to degraded QX200 ddPCR reagents). Amplitudes of all targets are lower and VIC+/Target + clusters (orange, brown, and pink) are not well-defined making data interpretation difficult. Panel **(C)** shows four 1D plots when multiplexing by optical channel using the Absolute Q dPCR system. *CYP2D6* targets and the *TERT* reference gene target are each labelled with unique florescent dyes (FAM, VIC, JUN, ABY). While the amplitude values of the positive reactions may overlap (FAM and ABY positive reactions cluster around 10,000 to 16,000 and VIC and JUN cluster around 2,000 to 5,000), the positive signals from each channel are able to be individually gated (blue background).

To identify complex *CYP2D6* structural variants including hybrid genes, simultaneous testing of multiple target regions allows for more accurate detection of such structures. The Association for Molecular Pathology (AMP) Pharmacogenetics Working Group, which provides guidance for clinical allele testing, recommends copy number testing in Tier 1 for deletions (CYP2D6*5) and duplications/multiplications (*xN*), and Tier 2 which includes hybrid genes such as CYP2D6*13 (Pratt et al., 2021). Multiplexing dPCR reactions can be accomplished two ways: 1) amplitude-based multiplexing where two assays with the same fluorescent dye are combined at different concentrations to achieve stratified clusters within a single optical channel, or 2) optical-based multiplexing, in which two or more assays with different fluorescent dyes are combined and analyzed in separate excitation-emission channels (Quan et al., 2018). Figure 2 illustrates amplitude-based multiplexing versus optical-based multiplexing. Amplitude-based multiplexing of two *CYP2D6* targets using the QX200 ddPCR system has been previously established as a reliable method for *CYP2D6* CN detection (Wang et al., 2022; Wen et al., 2022). Briefly, target assays with the same fluorescent dye, FAM^™^, are combined at 1.0x and 0.5x final concentrations so that signal amplitude at PCR-endpoint for the individual assays cluster together. “Positive” droplets for each target assay can then be easily segregated by amplitude-gating and independently calculated for CN determination. In contrast, optical-based multiplexing, a potential strategy for the Absolute Q Digital PCR system, can simultaneously interrogate up to four fluorescent targets: FAM/VIC/JUN/ABY versus FAM/VIC or FAM/HEX for the QX200 system. Of note, the same TaqMan copy number assay chemistries used for qPCR have been adapted for dPCR.

For CNV determination, dPCR platforms necessitate the fragmentation of genomic DNA (gDNA) for confluent sample disbursement and subsequent result reliability (Regan et al., 2015). Considerations for RE selection include target amplicon compatibility and methylation sensitivity. Specifically, for CNV analysis, RE digestion functions to separate target regions on the same DNA strand for proper distribution among microreactions (droplets or microchambers). Samples with greater than two copies of the target region or multiplexed reactions may be more sensitive to inefficient enzymatic digestions (Regan et al., 2015). In these cases, the calculated CN may be lower than expected and/or exhibit a loss in copy call clarity, e.g., 2.9 (rounds to 3) versus 2.5 (inconclusive). As such, strategic RE selection that fragments against multiple target copies or proximal targets of interest is pertinent for accurate CNV analysis. Conventionally, gDNA samples are pre-digested with RE(s) before adding to the PCR reaction mixture (Figure 1). Aliquots of samples are typically incubated and separately stored 1–24 h prior to testing. This pre-work step extends sample handling, increases the turn-around time and adds risk of user error. Furthermore, the use of intermediary aliquots may also limit the availability of sample for other testing or biobanking needs as digested gDNA may not be needed/suitable for other applications. Thus, it is advantageous to remove the pre-work step in efforts to simplify the analysis workflow, reduce sample-to-results time, and limit sample waste.

In this collaborative study, we developed methods of CN detection on the Absolute Q dPCR system and validated results against those previously generated by the QX200 ddPCR system. Our first aim was to validate the single-step “One-pot” RE digestion method for the Absolute Q. Next, we leveraged the multi-channel optical capabilities of the Absolute Q to multiplex three *CYP2D6* assays for CN determination targeting the 5′UTR, intron 6, and exon 9 regions. Third, CN results of DNA samples from different sources including blood, saliva, liver tissue, and the Coriell Institute were also compared with previous results that were generated by the QX200 ddPCR system. Finally, we also highlight how rare variation in reference genes can impact CN test results and interpretation.

## 2 Materials and methods

### 2.1 Samples

A total of 30 DNA samples that were previously identified as having CN calls of 0–6 were used in this study (Table 1). Thirteen DNA samples isolated from cell lines were obtained from the Coriell Institute for Medical Research (Camden, NJ, United States), of which six (NA24217, HG00463, NA19790, NA18959, NA19317, and NA17244) were also a part of the *CYP2D6* Genetic Testing Reference Materials Coordination Program (GeT-RM) (Gaedigk et al., 2019). Additionally, NA18933 has been extensively characterized by Wang et al. (2022). Nine of the DNA samples were extracted from human liver tissue; three were from the Liver Tissue Cell Distribution Center System (LTCDS) and six were from materials transferred from the Discovery Labware, Inc. (Corning Inc.) to Children’s Mercy (Kansas City, Missouri, United States) for research purposes. Eight DNA samples were from a repository maintained at the CMRI; four DNAs were isolated from saliva and four from whole blood collected in EDTA-containing vacutainers. The use of deidentified tissue and repository samples were approved by the Children’s Mercy Institutional Review Board.

**TABLE 1:** Summary of samples and their expected CN calls for the three interrogated *CYP2D6* target regions.

Sample ID	Consensus genotype	5′UTR	intron 6	exon 9
HG00139	*1/*2	2	2	2
HG00463^a^	*36+*10/*36+*10	4	4	2
HG00595^b^	*36+*10/*36x2+*10	5	5	2
HG02853	*29/*29x2	3	3	3
NA10836	*2/*68+*4	3	2	2
NA17111	*1/*1	2	2	2
NA17244^a^ ^,^ ^c^	*2x2/*4x2 (+hybrid)	4	5	4
NA18107^b^	*36x3+*10/*10x2	6	6	3
NA18933^d^	*5/*157	1	1	1
NA18959^a^	*2/*36+*10	3	3	2
NA19317^a^	*5/*5	0	0	0
NA19790^a^	*1/*13+*2	2	3	3
NA24217^a^	*2/*41x3	4	4	4
Liver-1	*1/*1	2	2	2
Liver-2	*1/*1	2	2	2
Liver-3	*1/*4N+*4	3	3	2
Liver-4	*1/*68+*4	3	2	2
Liver-5	*1/*5	1	1	1
Liver-6	*1x2/*41	3	3	3
Liver-7	*4/*17	2	2	2
Liver-8	*2/*2	2	2	2
Liver-9	*1/*1	2	2	2
Blood-1	*59/*68+*4	3	2	2
Blood-2	*1/*36+*10	3	3	2
Blood-3^b^	*5/*36x2+*10x2	4	4	2
Blood-4	*1x2/*4	3	3	3
Saliva-1	*1/*59	2	2	2
Saliva-2	*5/*10	1	1	1
Saliva-3	*1/*2	2	2	2
Saliva-4	*5/*5	0	0	0

^a^ Coriell sample is a part of the *CYP2D6* GeT-RM cohort (Gaedigk et al., 2019).

^b^ Diplotypes shown are the most likely; other SV arrangements are possible.

^d^ NA18933 was previously characterized (Wang et al., 2022).

^c^ NA17244 likely contains an additional hybrid gene copy; the structure of the allele harboring the hybrid has not been resolved.

DNA was extracted from liver, saliva, and whole blood using the DNeasy Blood and Tissue Kit (Qiagen, Hilden, Germany) per manufacturer’s protocol and quantified using a NanoDrop^™^ One instrument (Thermo Fisher Scientific, Waltham, MA, United States). Table 1 provides an overview of the samples, and their expected CN calls for each of the interrogated *CYP2D6* target regions (5′UTR, intron 6, exon 9). Expected CN calls refer to those obtained by orthogonal methods previously used for gene characterization as described below.

### 2.2 *CYP2D6* gene characterization

Consensus *CYP2D6* genotypes (Table 1) were informed by both copy number testing and genotyping using a combination of methods that have previously been described (Gaedigk et al., 2019; Boone et al., 2020; Wang et al., 2022; Wen et al., 2022; Turner et al., 2023). Briefly, single variant detection was performed with TaqMan genotyping assays in either single-tube format or custom OpenArray^™^ panels (Thermo Fisher Scientific, Waltham, MA, United States). Long-range PCR (XL-PCR) using gene-specific and allele-specific primers qualitatively captured *CYP2D6* structural variation including gene duplications, hybrids, and deletions. XL-PCR amplicons were visualized by agarose gel electrophoresis to inform structure, i.e., presence/absence of a duplication, deletion and/or hybrid and amplicon lengths. Selected XL-PCR amplicons were also subjected to Sanger sequencing with a 3730XL DNA Analyzer and BigDye^™^ Terminator v3.1 chemistry (Thermo Fisher Scientific, Waltham, MA, United States) to more fully characterize the structural variant (e.g., determine which portions of a hybrid were *CYP2D7*-derived).

### 2.3 CNV by QX200 ddPCR

Quantitative assessment of *CYP2D6* copy number by ddPCR was performed on the QX200 System (Bio-Rad, Hercules, CA, United States) using commercially available *CYP2D6* TaqMan copy number assays (Thermo Fisher Scientific, Waltham, MA, United States): 5′UTR (Hs04078252_cn), intron 6 (Hs04502391_cn), exon 9 (Hs00010001_cn), *TERT* (catalog no. 4403316), and RNaseP (catalog no. 4403326). The RE digest was performed by the traditional method (Figure 1), as a separate step prior to combining with QX200 ddPCR reagents: 50 ng gDNA was pre-digested according to the manufacturer’s protocol with restriction enzymes *EcoR*I-HF, *BamH*I-HF (New England Biolabs, Ipswich, MA, United States), or Anza 69 *Bgl*I (Invitrogen, Waltham, MA, United States), the appropriate supplied buffer, and nuclease-free water in a final volume of 20 µL.

CN status was determined by either testing a single *CYP2D6* locus or duplexed (two *CYP2D6* targets) by amplitude. Reactions for single *CYP2D6* targets were performed using a final concentration of 1.0x *CYP2D6* assay, 1.0x reference gene assay, 1x ddPCR Supermix for Probes (No dUTP), 15 ng of RE digested DNA, quantum satis (q.s.) with nuclease-free water to a final reaction volume of 21 µL. Reactions duplexed by amplitude were similarly combined, except one *CYP2D6* assay was added at 1.0x and the other at 0.5x to maximize cluster separation (Figure 2A). Reactions were transferred into a ddPCR 96-deep well plate, droplets generated with the QX200 AutoDG Droplet Generator and Droplet Generation Oil for Probes, heat-sealed with pierceable foil, and cycled in a C1000 Touch Thermal Cycler (initial denaturing and enzyme activation at 95°C for 10 min, 40x cycling at 94°C for 30 s and 60°C for 1 min, final enzyme deactivation at 98°C for 10 min, final droplet hardening at 4°C for 30 min, and hold at 10°C). Droplets were read with the QX200 Droplet Reader and results analyzed using the QuantaSoft™ Analysis Pro Software (version 1.0.596).

### 2.4 CNV by Absolute Q dPCR

Custom TaqMan gene copy assays were provided by Thermo Fisher Scientific (Waltham, MA, United States) and used for multiplexing on the Absolute Q as detailed in Table 2. The combination of 5′UTR, intron 6, exon 9, and *TERT* assays was used for most experiments while the combination of 5′UTR, intron 6, exon9, and RNaseP was used for follow-up testing on selected samples. Assays are referred to as “duplex” (2-plex) and “triplex” (3-plex) reactions because they inform two and three *CYP2D6* target regions, respectively, although these are technically 3-plex and 4-plex reactions due to also interrogating the reference gene.

**TABLE 2:** Summary of TaqMan copy number assays used to establish *CYP2D6* multiplexing on the Absolute Q. 5′UTR and exon 9 assays were used in every assay combination because they flank the *CYP2D6* gene region. The inclusion of intron 6 (or intron 2) provides additional information regarding the nature of a hybrid gene if present. All assay combinations included either *TERT* or RNaseP as the 2-copy reference.

Assay	Target region	Fluorescent dye	TFS derived assay ID^a^
5′UTR	*CYP2D6* 5′UTR	JUN	Hs04078252_cn
intron 2	*CYP2D6* intron 2	FAM	Hs04083572_cn
intron 6	*CYP2D6* intron 6	FAM	Hs04502391_cn
exon 9	*CYP2D6* exon 9	VIC	Hs00010001_cn
***TERT***	*TERT*	ABY	Cat # 4403316
RNaseP^b^	*RPPH1*	ABY	Cat # 4403328

^a^ TFS (Thermo Fisher Scientific)-derived Assay IDs, correspond to those with FAM (5′UTR, intron 2, intron 6, exon 9) or VIC (*TERT*, RNaseP) fluorescent dye labels. Alternative dye labels, such as those used in this study, are available per custom order.

^b^ The assay is referred to as RNaseP (gene name *RPPH1). RPPH1* encodes the H1 RNA component of ribonuclease P.

For testing the traditional digestion method on the Absolute Q, 100 ng of gDNA was incubated with 1 µL of 10 U/µL of Anza 69 *Bgl*I restriction enzyme, 1 µL of 10x Anza Clear Buffer (Invitrogen, Waltham, MA, United States), and q.s. with nuclease-free water for a total volume of 20 µL. The digest was gently combined, incubated at 37°C for 15 min, heat-inactivated at 80°C for 20 min and stored at −20°C until run on the Absolute Q (Thermo Fisher Scientific, Waltham, MA, United States). Reaction mixes consisted of 1x TaqMan assay mix (a duplex of 5′UTR and exon 9 with *TERT* as the reference assay), 1x Absolute Q DNA dPCR Mix, and q.s. with nuclease free water to 8 µL per reaction. Next, 2 µL of pre-digested genomic DNA was added the reaction mix. From this final mixture, 9 µL was loaded into each well of the MAP16 plate and subsequently layered with 15 µL of Absolute Q Isolation Buffer before loading on the Absolute Q instrument.

The single-step “One-pot” RE digestion method combined all reaction reagents into a single tube with final concentrations of 1 ng/μL undigested gDNA, 0.25 U/µL of Anza 69 *Bgl*I restriction enzyme, 0.25x Anza Clear Buffer, 1x TaqMan assay mix (duplex or triplex depending on the experiment), 1x Absolute Q DNA dPCR Mix, and q.s. with nuclease-free water for a total volume of 10 µL per reaction. Similar to the traditional digestion protocol, 9 µL of reaction mix and 15 µL of Isolation Buffer was added to each sample loading well. Prior to instrument loading, the plate was incubated at benchtop ambient temperature (20°C–25°C) for 30 min. A visual summary of both the traditional and One-pot workflows is provided in Figure 1. The “no RE digest” protocol was performed in the same manner as the One-pot, except the volume of reagents used for the RE digestion were replaced with nuclease-free water and no benchtop incubation was performed.

For all workflows, the MAP16 plate was then cycled on the Absolute Q instrument with the following protocol: preheat for 10 min at 96°C, followed by 40 cycles of denature (5 s at 96°C) and anneal/extend (15 s at 60°C). Data was analyzed using the Applied Biosystems™ QuantStudio™ Absolute Q™ Digital PCR Software (version 6.2.1).

### 2.5 Copy number variation (CNV) in *TERT* and RNaseP

The presence of copy number variation for two reference loci, *TERT* (GRCh38.p14 chr5:1288454-1292174:DEL) and *RPPH1* (RNAseP; GRCh38.p14 chr14:202343071-20343411), was assessed using the Progenetix tool, which analyzes 3,200 samples from the 1000 Genomes Project reference database for the presence of CNVs (Huang et al., 2021).

### 2.6 Data analysis

For both the Absolute Q dPCR and the QX200 ddPCR platforms, data plots were manually inspected for proper separation of clusters. A valid CN call was determined if the calculated copy number was within a threshold of 0.25 of an integer value, e.g., a CN of 2 was considered valid if the calculated value was between 1.75 and 2.25. Additionally for the Absolute Q, when the calculated CN values were outside of the threshold, the concentration of target copies (copies/µL) of each channel was assessed (ideally 100–500 copies/µL) to conservatively maintain linearity within the Poisson distribution assumption. If Absolute Q results returned more than 500 copies/µL, DNA was diluted, and the assay was repeated to ensure accuracy of copy number calculation (the measure of copies/µL may be impacted by the amount of DNA in the reaction, DNA purity/degradation, and/or copy number state of a sample). Samples with values outside the threshold ranges or inconsistent CN calls among target regions were also repeated using RNaseP reference assay in replacement of *TERT*. Absolute Q dPCR CN results were compared to those obtained from QX200 (Bio-Rad, Hercules, CA, United States) and consensus genotypes for validation.

## 3 Results

### 3.1 Restriction enzyme (RE) digestion workflows

Eight Coriell samples were tested on the Absolute Q with both the traditional and One-pot methods of restriction enzyme digestion (Figure 1). Calculated CN values are summarized in Table 3. While the calculated values vary slightly among the digestion methods, the traditional and One-pot methods yielded CN calls consistent with their expected calls (Table 1). Samples with CN calls greater than 2-copies indicate the RE digest efficiently cut between targeted *CYP2D6* regions in the presence of gene duplications and or hybrid genes. Additional experiments demonstrated effectiveness of the One-pot digestion method for template fragmentation from as early as 0 min to 72 h while conserving proper CN determination (Supplementary Figure 1).

**TABLE 3:** Comparison of the calculated CN values for Coriell DNAs treated with the traditional RE digestion workflow, the One-pot digestion workflow, and no RE digestion. All multiplex reactions were run with *TERT* as reference gene.

	Traditional RE		One-pot		No RE digestion	
Sample ID	5′UTR	exon 9	5′UTR	exon 9	5′UTR	exon 9
NA18933	1.04	1.00	1.04	1.05	—	—
NA17111	2.17	2.19	2.02	2.02	2.17	2.05
HG02853	3.25	3.15	3.00	2.99	2.65	2.58
NA24217	3.97	3.88	4.14	4.05	—	—
HG00463	4.03	1.99	4.05	2.04	3.22	2.14
NA19790	1.91	2.95	2.02	3.02	2.00	2.53
NA18959	3.04	2.08	2.98	2.03	—	—
NA10836	3.18	2.06	3.05	2.05	—	—

Four Coriell DNA samples were run without a RE treatment (Table 3). Undigested samples with expected CN calls >2 fell outside of the calculated CN thresholds indicating that target copies were not effectively separated. For example, HG02853 (3-copy) was run with the duplex assay (5′UTR, exon 9, *TERT*) resulted in calculated CN values of 2.65 (5′UTR) and 2.58 (exon 9), which is considerably lower than expected.

### 3.2 Multiplexing three CYP2D6 target regions with *TERT* and RNaseP reference assays

The eight Coriell samples assessed for the One-pot method (Table 3) were further used to develop and validate a *CYP2D6* triplex assay for the Absolute Q. Individual assays were combined into one reaction for the simultaneous interrogation of three *CYP2D6* regions: 5′UTR, intron 6, and exon 9 with a reference gene assay (*TERT* or RNaseP). Table 4 summarizes the calculated CN values from the assay combinations of 5′UTR, intron 6, exon 9, *TERT*; and 5′UTR, intron 6, exon 9, RNaseP on the Absolute Q. For both triplex assays, the calculated CN values for all samples were consistent with their expected CN calls (Table 1) and QX200 results (Table 4). An additional triplex combination targeting intron 2, as an alternative to the intron 6 target, was also tested. Results are summarized in Supplementary Table 1. While this triplex combination also performed well, it was not pursued for further assay development.

**TABLE 4:** Calculated CN calls for two triplex assay combinations on the Absolute Q compared to the calculated CN calls obtained on the QX200 platform. 5′UTR, intron 6 and exon 9 refer to assays targeting those respective *CYP2D6* regions. Triplex reactions were combined with *TERT* or RNaseP reference gene assays.

	Absolute Q						QX200		
	Reference: *TERT*			Reference: RNaseP			Reference: *TERT*		
Sample ID	5′UTR	intron 6	exon 9	5′UTR	intron 6	exon 9	5′UTR	intron 6	exon 9
NA18933	0.94	0.98	0.98	1.01	1.02	1.04	0.98	0.91	1.14
NA17111	2.10	2.10	2.11	2.02	2.04	2.05	2.01	2.14	2.02
HG02853	3.20	3.23	3.21	3.04	3.07	3.04	3.01	2.98	3.13
NA24217	3.92	4.01	4.02	4.11	4.17	4.10	3.87	3.94	4.04
HG00463	4.10	4.12	2.12	4.09	4.16	2.08	3.95	3.91	2.07
NA19790	2.06	3.09	3.12	2.08	3.09	3.06	2.07	3.00	3.15
NA18959	3.14	3.19	2.15	3.05	3.06	2.05	3.01	2.84	2.07
NA10836	3.05	2.05	2.02	3.09	2.12	2.08	2.90	1.99	2.03

### 3.3 Cross-platform validation using samples from different DNA sources

Twenty-two DNA samples (n = 10, liver tissue; n = 4, blood; n = 4, saliva, and n = 4, additional DNAs from Coriell) were tested using the triplex assay with *TERT* on the Absolute Q. The calculated CN values, ranging from 0–6 copies, were also compared to results from the QX200 (Tables 5, 6). Table 5 summarizes 18 samples that passed validation criteria, i.e., met the calculated CN threshold (within 0.25 of the integer value), had proper separation of reaction clusters on the scatter plots, the concentrations were between 100 and 500 copies/µL, and CN calls were in alignment with the consensus genotype. The DNA samples isolated from liver, blood, saliva, and those from Coriell represented 0–5 copies. Of note, the result for the 5′UTR target obtained on the QX200 platform for sample Blood-3 was outside of our established CN threshold (calculated CN value of 3.56). However, since the Absolute Q result was within the threshold (calculated CN value of 4.01) and the 5′UTR 4-copy call was consistent with the sample’s genotype (CYP2D6*5/*36x2+*10x2), the Absolute Q results were considered valid.

**TABLE 5:** Comparison of calculated CN values for the Absolute Q and QX200 platforms. The multiplex assay for the Absolute Q contained all three *CYP2D6* targets and *TERT*. Data obtained on the QX200 were either from single-target reactions or 5′UTR and exon 9 duplexes with *TERT*. 5′UTR, intron 6 and exon 9 refer to assays targeting those respective *CYP2D6* regions. Results shown are for the 18 samples which initially met validation criteria; data for an additional eight samples are provided in Table 4. Data for the remaining five samples are provided in Tables 6, 7 as theses underwent additional testing.

	Absolute Q			QX200		
Sample ID	5′UTR	intron 6	exon 9	5′UTR	intron 6	exon 9
Blood-1	2.86	2.00	2.04	2.78	1.92	2.02
Blood-2	3.04	3.07	2.05	2.81	3.00	2.08
Blood-3	4.01	4.19	2.06	3.56	4.06	1.89
Blood-4	3.04	3.10	3.05	2.71	2.67	2.88
Saliva-1	2.03	2.14	2.18	1.89	2.07	2.12
Saliva-2	1.00	1.16	1.16	0.91	0.93	1.15
Saliva-3	1.91	2.13	2.13	1.83	2.15	2.21
Saliva-4	0.01	0.01	0.00	0.00	0.01	0.02
Liver-1	2.01	2.01	2.01	1.94	ND	1.95
Liver-2	1.85	1.89	1.87	1.90	1.87	2.10
Liver-3	2.97	3.03	2.02	3.16	3.00	1.96
Liver-3	2.95	2.01	1.98	2.85	2.01	2.14
Liver-5	1.04	1.03	1.04	0.99	0.93	0.94
Liver-6	2.99	2.94	2.96	2.97	2.89	2.99
Liver-8	1.92	1.96	1.96	2.01	1.99	1.91
HG00595	5.02	4.98	2.03	4.99	5.04	1.97
NA17244	3.96	5.13	4.11	4.09	4.71	4.24
NA19317	0.01	0.00	0.00	0.00	0.00	0.00

**TABLE 6:** Comparison of samples Liver-7, Liver-9, NA17244, and NA18107 across platforms and treatments. Treatments include sample dilutions as indicated in the “Aliquot” column, and the triplex assay used, where 5′UTR, intron 6, and exon 9 were combined with either *TERT* or RNaseP, as indicated in the “Reference” column. The number of copies/µL detected are indicated in parenthesis for each target region for the Absolute Q generated data. Samples analyzed with the QX200 platform were either run in single-target reactions or 5′UTR and exon 9 assays were duplexed by amplitude.

		Absolute Q				QX200		
Sample ID	Aliquot	Reference	5′UTR	intron 6	exon 9	5′UTR	intron 6	exon 9
Liver-7.1	Original	*TERT* (524)	1.99 (520)	2.20 (575)	2.30 (604)	3.40	2.21	4.02
Liver-7.1	1:1 Dilution	*TERT* (248)	2.04 (253)	2.15 (267)	2.30 (286)	1.88	2.20	2.20
Liver-7.1	1:1 Dilution	RNaseP (176)	2.66 (233)	2.99 (263)	3.04 (267)	2.74	2.93	3.08
Liver-7.2	Original	*TERT* (231)	1.94 (224)	1.96 (227)	1.98 (229)	1.80	1.83	1.77
Liver-7.2	Original	RNaseP (219)	2.08 (228)	2.13 (234)	2.13 (234)	1.86	1.91	1.86
Liver-9	Original	*TERT* (568)	2.10 (595)	2.03 (577)	2.07 (589)	2.33	2.35	2.29
Liver-9	1:3 Dilution	*TERT* (125)	2.17 (135)	2.11 (131)	2.17 (135)	—	—	—
NA17244	Original	*TERT* (128)	3.96 (253)	5.13 (328)	4.11 (262)	4.09	4.71	4.24
NA17244	Original	RNaseP (142)	3.80 (269)	4.76 (334)	3.89 (275)	—	—	—
NA18107	Original	*TERT* (224)	6.28 (702)	6.38 (714)	3.10 (347)	5.15	6.01	2.94
NA18107	1:2 Dilution	*TERT* (97)	5.82 (281)	5.91 (286)	3.01 (145)	—	—	—

Four samples were subjected to follow-up testing summarized in Table 6. Liver-7.1, Liver-9 and NA18107 failed the validation criteria, while NA17244 was selected for additional confirmatory testing. Liver-9 had calculated CN values that were not within the criteria range and copies/µL exceeded 500. Diluting the gDNA of this sample 1:3 decreased the number of copies/µL and produced a calculated CN value within our set threshold parameters. Similarly, NA18107 had an initial high concentration of copies/µL for the 5′UTR and intron 6 assays of 702 and 714 copies/µL, respectively, which likely pushed the calculated CN values outside of our threshold. The issue was resolved by diluting DNA 1:2 and repeating the run.

Sample Liver-7.1 also had calculated CN values outside of the threshold on both platforms. The initial calculated CN on the Absolute Q differed considerably from that obtained by the QX200. To address this discrepancy, DNA preparation Liver-7.1 was diluted 1:1 and repeated with both *TERT* and RNaseP reference assays. A higher-quality DNA preparation (Liver-7.2) was also tested with *TERT* and RNaseP to assess whether DNA quality contributed to the inconsistent CN values. On both platforms, the calculated CN values obtained with the higher-quality Liver-7.2 DNA preparation indicated that this sample is indeed copy-neutral, i.e., has 2-copies for all *CYP2D6* target regions. The results from using either *TERT* or RNaseP also matched the consensus genotype (CYP2D6*4/*17). Furthermore, when Liver-7.1 DNA preparation was diluted 1:1, the CN value also resulted in a 2-copy call on the Absolute Q, although the exon 9 result remained outside the threshold (2.30). It is important to note that the 1:1 dilution preparation of Liver-7.1 had noticeably different copies/µL for each of the reference genes, 248 copies/µL for *TERT* and 176 copies/µL for RNaseP, even though they were both run with the same amount of DNA. The difference in copies between *TERT* and RNaseP skewed the calculated CN value for each respective run, even though the number of copies/µL for the *CYP2D6* target regions were similar (5′UTR at 253 vs. 233, intron 6 at 267 vs. 263, and exon 9 at 286 vs. 267 copies/µL). Such a difference in copies/µL was not observed for the reference genes for sample Liver-7.2 where 231 copies/µL were detected for *TERT* and 219 copies/µL for RNaseP.

NA17244 is a known structurally complex sample from the *CYP2D6* GeT-RM project and was thus followed up with additional testing. Reported CN calls are 4, 5 and 4-copies for the 5′UTR, intron 6, and exon 9 target regions, respectively (Gaedigk et al., 2019). Although NA17244 passed our validation criteria (Table 5), it was run with both *TERT* and RNaseP reference gene assays to explore the possibility of variant(s) within these reference gene(s) impacting assay results. NA17244 had not previously been tested with RNaseP on a dPCR platform. Results from both the initial triplex run and follow-up testing with RNaseP are shown in Table 6 for comparison and are consistent with previous reports regardless of reference gene used. This finding substantiated the conclusion of the GeT-RM study authors that one of the alleles likely contain an additional hybrid gene copy.

### 3.4 CNV identified in *TERT* reference gene

Sample HG00139 (consensus genotype CYP2D6*1/*2) also failed validation criteria due to CN values being outside of the threshold at all three targeted regions when using *TERT* as the reference gene assay (Table 7). Furthermore, a duplication or deletion was previously not detected by XL-PCR (Table 1). When the sample was retested with RNaseP as reference, the calculated CN indicated 2-copy at all *CYP2D6* interrogated regions. A search of the 1000 Genomes reference sample cohort with the Progenetix tool identified a duplication of *TERT* for HG00139 (3 copies). Subsequent testing of *TERT* copy number status using RNaseP as the reference gene confirmed the *TERT* 3-copy status identified by the Progenetix tool.

**TABLE 7:** HG00139^a^ was tested with three different assay combinations. Combination 1: RNaseP, *TERT*; Combination 2: 5′UTR, intron 6, exon 9, *TERT*; Combination 3: 5′UTR, intron 6, exon 9, RNaseP. The number of copies/µL are displayed next to the interrogated region in parenthesis. Because both assayed regions for combination 1 are typically used as reference gene targets, a different assay combination strategy was utilized to segregate the two targets. The commercially available *TERT* reference assay (Thermo Fisher Scientific; catalog no. 4403316), labeled with VIC, was combined with the RNaseP reference assay, which was customized with ABY for this study.

Assay combination #	Reference	Target	Calculated CN values
1	RNaseP (148)	*TERT* (218)	2.98
2	*TERT* (213)	5′ UTR (156)	1.46
2	*TERT* (213)	intron 6 (147)	1.38
2	*TERT* (213)	exon 9 (150)	1.41
3	RNaseP (151)	5′UTR (146)	1.94
3	RNaseP (151)	intron 6 (144)	1.91
3	RNaseP (151)	exon 9 (147)	1.95

^a^ This sample was identified using the 1000 Genomes data cohort with the Progenetix tool (https://progenetix.org/progenetix-cohorts/oneKgenomes/; last accessed 27 June 2024) (Huang et al., 2021).

Table 7 summarizes the results for HG00139. Assay combination 1 (interrogation of *TERT*, using RNaseP as reference) conclusively identified 3 copies for *TERT* (calculated CN: 2.98). Assay combinations 2 and 3 (triplex assays with *TERT* or RNaseP reference assays, respectively) exemplify the effect of 3 copies of *TERT* when testing for *CYP2D6* copy number versus a normal 2-copy RNaseP reference. The ratio of target to reference is 2:3 in assay combination 2, which proportionally skews the calculated CN value to fall outside of the 0.25 threshold. In assay combination 3, the ratio is 1:1 as expected, and the calculated CN value is within the threshold for each *CYP2D6* target.

Further evaluation of the 1000 Genomes cohort using the Progenetix tool also identified a *TERT* deletion in sample HG02756. However, this sample is no longer available through Coriell and could therefore not be experimentally confirmed. There were no samples among the interrogated 1000 Genomes cohort with copy number variation for *RPPH1* (RNAseP).

## 4 Discussion

Reliable detection of *CYP2D6* SV/CNVs is essential for the accurate prediction of a patient’s metabolizer status, or phenotype, to inform drug therapy. While qPCR has conventionally been used for CN detection, dPCR has emerged as a superior method due to the application of Poisson statistics for absolute target quantitation, allowing for the mitigation of common factors that can influence PCR amplification such as the presence of inhibitors and primer-template mismatches. Additionally, dPCR can resolve higher order of copy number states (>3-copies) (Whale et al., 2016). In this study, we validated a new dPCR platform, the Absolute Q, for *CYP2D6* CN detection by comparing to results previously generated by the QX200 ddPCR system. Additionally, the performance of the single-step RE digestion protocol (the One-pot method) and multiplexing three *CYP2D6* targets by optical channel on the Absolute Q instrument were verified. We also discovered a sample with a duplication of *TERT*, a commonly used reference gene for CN determination. Knowing how variation in a reference gene can impact assay results is an instrumental part of CN data interpretation.

Next-generation sequencing based methods have vastly improved over past years with long-read technologies being especially relevant for SVs/CNVs detection. These methods are, however, still more costly, require more expensive instrumentation and computational support compared to targeted testing. Data analysis is also more complex as targeted testing is limited to interrogating selected informative SNVs and copy number targets. Multiplexing as presented in this report is therefore an attractive approach to accurately determine multiple target regions in a single reaction to minimize cost and effort and increase sample turn-around times. This approach can also easily be adapted to improve testing of SVs/CNVs of other pharmacogenes such as *CYP2A6* or other genes of interest.

While assaying one *CYP2D6* gene region may satisfy the minimum Tier 1 AMP recommendations, certain structures elude detection when only one gene region is targeted. For example, CYP2D6*13+*2/*1 can only be detected when two *CYP2D6* gene regions such as the 5′UTR (CN = 2) and exon 9 (CN = 3) are interrogated. CYP2D6*13 is a *CYP2D7::CYP2D6* hybrid gene that can occur in a duplication arrangement upstream of a *2, as illustrated in this example, but also on its own as a “singleton” gene copy. The CN imbalance of interrogating 5′UTR and exon 9 here is due to CYP2D6*13 having a *CYP2D7*-derived exon 1 sequence that renders it nonfunctional. If only the 5′UTR region is tested, this sample would yield a CN call of 2 indicating the presence of two gene copies predicting normal metabolism. While the hybrid gene would not be identified, this phenotype prediction is correct as *13 is nonfunctional. In contrast, testing the exon 9 region only produces a CN call of 3 indicating the presence of a gene duplication and an incorrect ultrarapid phenotype assignment. However, having CN calls for both the 5′UTR and exon 9 regions reveals the presence of a *13 hybrid gene. Therefore, testing at least two, if not three or more, regions for copy number variation increases confidence in overall CN calls, allows more precise detection of the many structural variants and thus, phenotype classification. Testing for introns 2 or 6 may results in a CN call of 2 or 3 depending on whether this gene region is *CYP2D6* or *CYP2D7*-derived. For additional examples and details please see the PharmVar Tutorial on *CYP2D6* Structural Variation Testing and Recommendations on Reporting (Turner et al., 2023).

For *CYP2D6*, a limitation of both quantitative methods, qPCR and dPCR (QX200, Absolute Q or other platform), is the inability to detect a structural variation if there is no change in copy number (i.e., the sample is copy-neutral at CN = 2). An example is CYP2D6*2/*2 versus CYP2D6*5/*2x2. The former has a two-copy state where *CYP2D6* exists as one copy on each allele while the latter has structural variants where *CYP2D6* is deleted on one chromosome (*5) and the other has a *2x2 gene duplication. However, based on current knowledge, both diplotypes translate to the same phenotype, i.e., normal metabolizer. If it is deemed necessary to distinguish these, other methods need to be applied, which are discussed in detail in the PharmVar *CYP2D6* CNV tutorial (Turner et al., 2023).

### 4.1 Absolute Q validation

Validation included the One-pot digestion method for the Absolute Q. In this method, the RE digestion was combined with gDNA and dPCR reagents in a single reaction mix (Figure 1). We demonstrated that the One-pot protocol effectively digested the DNA. Because this method saves time and consumables, it was used for all CN experiments on the Absolute Q. Viability experiments for the One-pot RE digest were carried out by Thermo Fisher prior to this study and are available in the Supplementary Materials. These experiments assessed the efficacy of a dedicated benchtop incubation period after RE addition. A 0-min incubation period, i.e., no dedicated benchtop incubation, was found to yield CN results comparable to the 30-min incubation period used in this study. While 0-min RE incubations yielded valid results, a separate experiment comparing the addition or omission of a RE determined that the addition of an RE is necessary for overall copy number determination. As shown in Table 3, targets above 2-copies performed without RE digestion were either not consistent with the expected CN calls and/or fell outside of the 0.25 threshold. This suggested that the assay targets could not be properly separated via the MAP16 compartmentalization process-induced mechanical shearing of the DNA alone. These findings underscore the importance of effective RE digestion for accurate CN detection, particularly for samples with higher CNs when tested with multiplexed assay targets.

Since the *CYP2D6* gene locus may present with complex structural variants, interrogating multiple gene regions is required for comprehensive analysis and accurate diplotype calls. However, with each additional target region, the amount of effort and cost of consumables increases. The multiplexing method presented here allows for the simultaneous testing of up to three *CYP2D6* target regions (5′UTR, intron 6, and exon 9), which more efficiently determines *CYP2D6* copy number status in a simple workflow. To multiplex by optical channel on the Absolute Q, each target was labeled with a unique florescent dye and gated on multiple scatter plots (one for each optical channel). Resulting calculated CN values were comparable to results from the QX200 system, which were generated by either single-target reactions or multiplexed by amplitude. Overall, the Absolute Q demonstrated consistent results across samples regardless of CN status (0–6 copies) and DNA source (Coriell, blood, saliva, liver tissue).

Challenging sample types, specifically liver tissue DNA, were tested to assess the limitations of sample quality with this methodology. Liver tissue DNA can often be co-purified with inhibitors due the ease of overloading extraction columns or beads with crude material. The condition of the source tissue, e.g., prolonged time before freezing or processing, can also affect accurate CN determination. DNA samples Liver-7.1 and Liver 7.2 were of particular interest as these demonstrated the impact of poor-quality DNA for CN determination. Liver-7.1 DNA integrity was assessed by agarose gel electrophoresis which revealed substantial degradation (data not shown). A notable observation from these two samples is the increase of approximate 1-copy of *CYP2D6* attributed to the difference of copies of RNaseP vs *TERT* references. This large difference is unlikely due to operator variability such as pipetting because the copies/µL detected for the other target regions were similar between the two runs (Table 7). A deletion of RNaseP or a duplication of *TERT* was also excluded since the higher quality DNA preparation (Liver-7.2) resulted in consistent CN values for both reference genes on both platforms. Thus, substantial DNA degradation most likely contributed to the ambiguous CN calls for the Liver-7.1 preparation, as the degradation state may affect the test and reference gene loci to different extents, causing inconsistent assay results.

After assessing samples representing the most commonly observed SVs/CNVs (e.g., CYP2D6*2x2, *4x2, and *5 yielding 0–4 copies) additional Coriell samples with higher and more complex SVs/CNVs were tested. Calculated CN values may exhibit a drop in call clarity at expected target-to-reference ratios greater than 5. For example, a 10% difference in quantification would not impact a 2-copy sample results but may skew an 8-copy sample result to an ambiguous call. In these scenarios, call clarity can be restored by repeating with a 1:2 or 1:3 dilution of the initial sample input. For the CNs of 6 for 5′UTR and intron 6 target regions of NA18107 (Table 6), issues with call clarity were more evident. The initial triplex assay produced over 500 copies/µL (702 and 714 copies/µL for the 5′UTR and intron 6 targets, respectively). This caused an increase in the calculated CN values pushing them outside of the threshold. Diluting the DNA to produce below 500 copies/µL resolved the high copy number call.

Given the high degree of homology between *CYP2D6* and its pseudogene *CYP2D7*, it is important to ensure that assays are gene-specific, and all signals are exclusively generated from the intended target gene, i.e., *CYP2D6*. NA19317 has a *CYP2D6* deletion on both alleles (CYP2D6*5/*5), and thus CN calls of 0-copy demonstrated that the assays were indeed specific when performed under protocol conditions. A CN call of 0-copy was also obtained for a second CYP2D6*5/*5 sample, Saliva-4.

Coriell sample NA17244 was investigated because it had been extensively characterized for *CYP2D6* by multiple laboratories within the GeT-RM project (Gaedigk et al., 2019). The Absolute Q CN results were consistent with the GeT-RM, which was determined from multiple platforms to have 4 copies at the 5′UTR, exon 1, and intron 2, 5 copies at the intron 5 and intron 6, and 4 copies at exon 9 regions. Although, the specific configuration of the SV/CNV in this sample has not been fully resolved (consensus GeT-RM genotype: CYP2D6*2x2/*4x2 +hybrid), it is speculated that an additional hybrid gene copy is likely causing the observed CN pattern. Testing NA17244 in this study was not meant to elucidate the structural arrangement, rather, we sought to demonstrate the imbalanced CN calls were not due to the choice of reference gene and to confirm the previous published CN calls.

### 4.2 Considerations for the reference assay

Gene copy number variation in *TERT* has been documented in cancer cases (Kutilin et al., 2019; Choi et al., 2021; McKelvey et al., 2021) where gene amplifications of *TERT* were observed in somatic tissue samples. However, copy number variation has not been systematically reported or extensively interrogated in the germline DNA of healthy individuals. It is unknown whether the *TERT* gene duplication in HG00139 is a result of the cell line immortalization process or whether this is a rare germline event. However, the discrepancy from CN testing was resolved by repeating the dPCR assays using RNaseP.

Based on our findings, RNaseP (*RPPH1* gene) should be favored over *TERT* to avoid erroneous or ambiguous CN calls due to rare copy number events afflicting *TERT*. However, rare variants in RNaseP have also been reported to impact CN testing (Sicko et al., 2022). A strategy to avoid issues based on variation in reference genes may be parallel testing with two or more reference genes or develop higher-plex assays that allows multiple reference genes to be incorporated. Additionally, as another option, the RNaseP reference assay may be redesigned to avoid SNP interference.

## 5 Conclusion

For CN determination, the Absolute Q may detect up to three unknown target regions, while having one channel reserved to detect the reference assay. While amplitude and optical channel methods of multiplexing, can be effectively utilized, amplitude multiplexing may be more limited by the efficiency of the PCR reactions. Suboptimal reagent or input DNA may lead to the merging of clusters, thus the inability to separate individual targets (Whale et al., 2016). Multiplexing by optical channel does not have this problem, as targets are labeled with different fluorescent dyes and thus, are measured on different excitation-emission wavelengths. However, this method does require an instrument with multichannel capabilities (More than FAM and VIC) and assays with custom dyes, which currently are not available “off-the-shelf” and require custom ordering.

In this study, the Absolute Q dPCR system yielded *CYP2D6* CN calls that were comparable to those previously obtained with the QX200 ddPCR system. Copy number calls of both systems were consistent with their consensus *CYP2D6* genotypes, which are based on extensive testing. The One-pot digestion method and optically multiplexing three *CYP2D6* target regions facilitates the time-effectiveness in testing without compromising assay accuracy. While dPCR is a robust method following a straight-forward and scalable workflow, rare variation in reference genes, high copy number, and sample quality are factors that must be considered when performing and evaluating CN experiments.

## Data Availability

The original contributions presented in the study are included in the article/Supplementary Material, further inquiries can be directed to the corresponding author.
