# Supplementary material for: CYP2D6 copy number determination using digital PCR
Source: Front Pharmacol. 2024 Aug 14;15:1429286. doi: 10.3389/fphar.2024.1429286 (PMC11349684; doi:10.3389/fphar.2024.1429286)
Supplement: Supplementary file 1 [file DataSheet1.docx]

Supplementary Material

*CYP2D6* Copy Number Determination using Digital PCR

Wendy Y Wang, Lancy Lin, Erin C Boone, Junko Stevens, Andrea Gaedigk *

*** Correspondence:** Andrea Gaedigk, agaedigk@cmh.edu


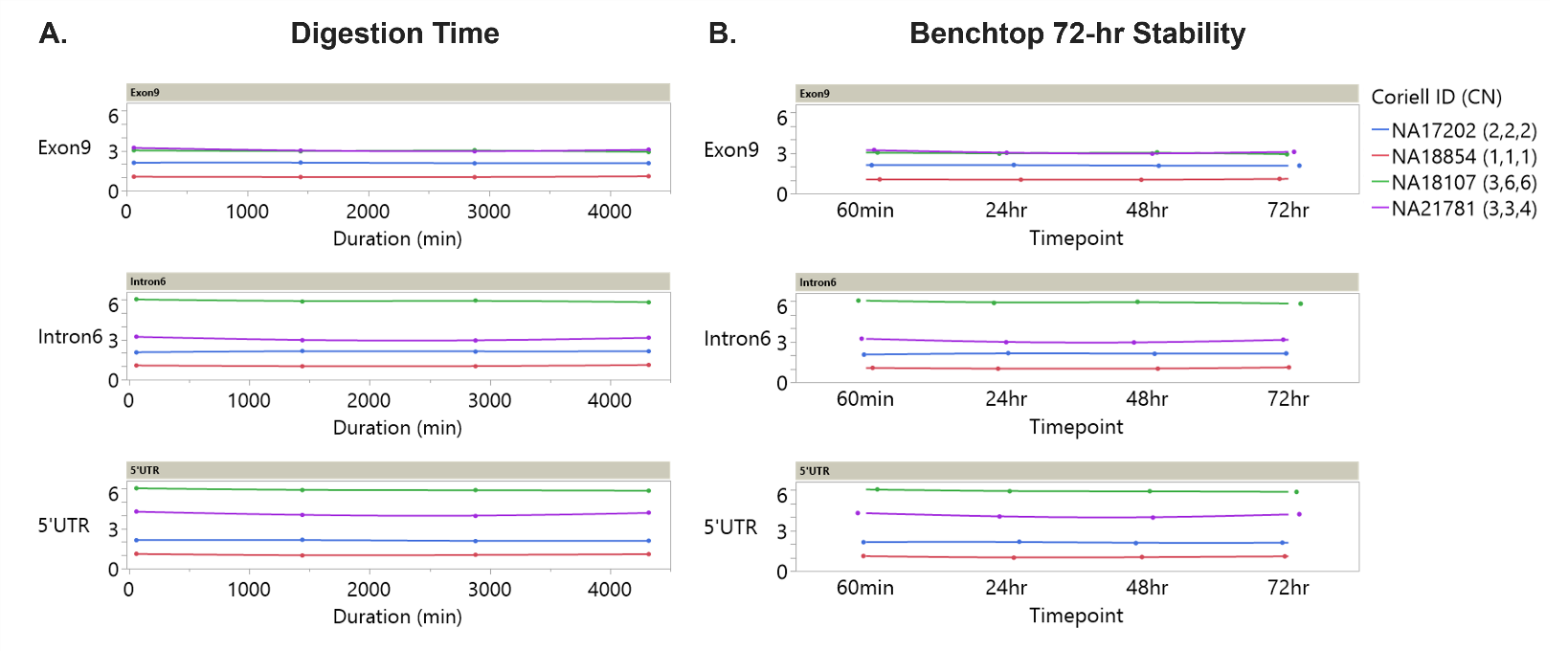


**Supplementary Figure 1.** Benchtop usability data of the One-pot ambient enzymatic digestion method. Four Coriell gDNA samples with *CYP2D6* CNs were digested using the *Bgl*I enzyme and then tested with the multiplex 5’UTR, intron 6, exon 9, and *TERT* assays. Digestion time began upon loading the assembled reaction mixtures into the MAP16 sample loading wells. A) Digestion Efficacy: durations ranging from 0 mins (i.e. direct-to-instrument, no latency period) to 60 mins were assessed for copy calls. Results were found to be concordant to pre-digest conditions as early as 0 mins. 30 mins was selected as the standard digestion time for data repeatability. B) Benchtop Stability: samples were assessed over a 72-hr period for over-digestion. Most samples retained N±0.3 copy call clarity, except for one datapoint (CN=6.35). Since high copies of *2D6* are rare, 72-hr stability can be claimed for the majority of samples.

**Supplementary Table 1.** Multiplexing *CYP2D6* target regions 5’UTR, intron 2, and exon 9 with reference assays *TERT* and RNaseP on the Absolute Q. In addition to intron 6, intron 2 is also a common locus to target within the *CYP2D6* gene region. This triplex combination was not further tested in this study.

|  | *TERT* | | | RNaseP | | |
| --- | --- | --- | --- | --- | --- | --- |
| Sample ID | 5'UTR | intron 2 | exon 9 | 5'UTR | intron 2 | exon 9 |
| NA18933 | 1.12 | 1.07 | 1.11 | 1.01 | 0.91 | 1.05 |
| NA17111 | 2.10 | 2.03 | 2.03 | 2.13 | 2.02 | 2.03 |
| HG02853 | 3.27 | 3.18 | 3.24 | 3.29 | 3.16 | 3.22 |
| NA24217 | 4.04 | 3.86 | 4.04 | 4.00 | 3.74 | 4.06 |
| HG00463 | 3.94 | 3.76 | 1.94 | 3.85 | 3.74 | 1.86 |
| NA19790 | 1.96 | 2.05 | 3.03 | 2.01 | 1.93 | 2.98 |
| NA18959 | 3.14 | 3.06 | 2.06 | 2.85 | 2.72 | 1.97 |
| NA10836 | 3.11 | 2.06 | 2.09 | 3.29 | 2.08 | 2.15 |
